# Inferring microbial interaction network from microbiome data using RMN algorithm

**DOI:** 10.1186/s12918-015-0199-2

**Published:** 2015-09-04

**Authors:** Kun-Nan Tsai, Shu-Hsi Lin, Wei-Chung Liu, Daryi Wang

**Affiliations:** Biodiversity Research Center, Academia Sinica, Taipei, 115 Taiwan; Department of Medical Research and Development, Show Chwan Health Care System, Changhua, 505 Taiwan; Institute of Statistical Science, Academia Sinica, Taipei, 115 Taiwan

**Keywords:** Microbiome, Next-generation sequencing, Microbial regulatory network, Cooperative and competitive relationship, OTU-triplet model, RMN algorithm

## Abstract

**Background:**

Microbial interactions are ubiquitous in nature. Recently, many similarity-based approaches have been developed to study the interaction in microbial ecosystems. These approaches can only explain the non-directional interactions yet a more complete view on how microbes regulate each other remains elusive. In addition, the strength of microbial interactions is difficult to be quantified by only using correlation analysis.

**Results:**

In this study, a rule-based microbial network (RMN) algorithm, which integrates regulatory OTU-triplet model with parametric weighting function, is being developed to construct microbial regulatory networks. The RMN algorithm not only can extrapolate the cooperative and competitive relationships between microbes, but also can infer the direction of such interactions. In addition, RMN algorithm can theoretically characterize the regulatory relationship composed of microbial pairs with low correlation coefficient in microbial networks. Our results suggested that *Bifidobacterium*, *Streptococcus*, *Clostridium XI*, and *Bacteroides* are essential for causing abundance changes of *Veillonella* in gut microbiome. Furthermore, we inferred some possible microbial interactions, including the competitive relationship between *Veillonella* and *Bacteroides*, and the cooperative relationship between *Veillonella* and *Clostridium XI*.

**Conclusions:**

The RMN algorithm provides the reconstruction of gut microbe networks, and can shed light on the dynamical interactions of microbes in the infant intestinal tract.

**Electronic supplementary material:**

The online version of this article (doi:10.1186/s12918-015-0199-2) contains supplementary material, which is available to authorized users.

## Background

Microbes are the most abundant and diverse organisms on earth and their interactions are crucial in understanding both the ecology and the evolution of microorganisms. Microbial interactions, including mutualism, competition, parasitism and commensalism, are difficult to quantify as the underlying processes usually cannot be observed directly and are often too complex for laboratory experiments [1]. However, recent advances in high-throughput sequencing technology have made large scale surveys of microbial communities feasible. Metagenomic studies and network-based approaches have yield detailed information on the composition of microbial communities, which in turn pave the way to study the structure of microbial ecosystems and their dynamics [2–4].

Elucidating competitive and cooperative relationship is a challenge in generating a microbial interaction network because of the direction of such interactions [5]. Competition and cooperation are the two most studied microbial interactions in the recent times with the former dominating the latter in various microbial communities [6–9]. Recent studies have also shown that competitive interactions can drive the evolution of cooperation in microbial ecosystems [9]. Therefore, identifying competitive and cooperative relationships between microbes is profound importance; however, the directional nature of such interactions also poses as a difficult challenge in network construction.

There are several approaches in constructing a microbial network. One commonly used method is the similarity-based network construction where the co-occurrences of two species over multiple time-series samples are measured to infer their interaction [2, 3, 10–12]. In such networks, nodes correspond to organisms and an edge between two nodes represents the significant relationship of two taxa across a set of time series samples. Although other derived approaches [13–15] can identify the pairwise relationships between microbes by using correlation estimation, they do not identify the nature and the strength of these relationships [1]. In general, these methods cannot capture the complexity of microbial interactions and cannot elucidate how microbes regulate each other.

To model complex relationships (one species influencing multiple others), methods employing mathematical and statistical models have been developed recently. For example, the generalized Lotka–Volterra framework was used to model the dynamics of microbial populations in order to estimate parameters governing species-species interactions [16–18]. Moreover, other studies have used nonparametric regression models to infer the dynamic relationships between three microbial populations [19, 20]. These regression models use smooth functions to describe microbial relationships and estimate their relative effects. Because these regression analyses do not use *a priori* knowledge to build dynamic microbial interactions, the prediction from those models often lack biological explanations, especially when the microbial community under examination is a complex one [21]. Therefore, how to construct a meaningful microbial interaction network remains a challenge in microbial ecology.

Due to the importance of competitive and cooperative relationships between microbes in complex ecological communities [8, 22, 23], an inference model for constructing microbial regulatory network should consider these interactions simultaneously. However, all of the studies mentioned above do not incorporate these features into their construction of regulatory networks. Association rule mining can be a promising way to infer complex relationships. A rule-based method, named smooth response surface (SRS) algorithm, was developed to construct complex regulatory networks [24]. Basing on a piecewise linear-quadratic polynomial, the SRS algorithm uses a three-dimensional model surface (3D-MS) to successfully infer the relationships between the target, activator, and repressor nodes.

In this paper, we adopt a similar rationale as the SRS algorithm to detect the cooperative and competitive relationships between microbes for constructing microbial regulatory networks. Our new methodology, named the rule-based microbial network (RMN) algorithm, is an advanced version of the rule-based approach. We used Tanh functions for modeling nonlinear responses as regulatory effects of microbes. To our understanding, Tanh functions have been used to model nonlinear responses in some microbial studies, such as dose influences of pathogens [25], regulatory effects of microbes [26], influences of microbial compositions (phospholipids fatty acids) [27]. The RMN algorithm uses a nonlinear regulatory OTU-triplet (NRO) model to capture cooperative and competitive relationships and consequently infers the directional interaction between microbes. We applied our new approach to simulated data and showed that the RMN algorithm was capable to reconstruct microbial regulatory networks. Furthermore, we used Koenig’ data (2011) to construct the interaction network for the microbial community in infant gut [28]; and our results suggest that *Bifidobacterium*, *Streptococcus*, *Clostridium XI*, and *Bacteroides* play essential roles in regulating the abundance of *Veillonella* in an infant gut microbiome.

## Methods

### Sample collection

To construct microbial regulatory networks in infant intestinal tracts, we used the pyrosequencing reads of 16S rRNA genes from 61 infant gut samples, which were treated with four diet states at four time periods [28]. These infant gut samples were collected over 61 time points. The average length of 16S rRNA gene sequences were 237 bps [28], we used only 16S rRNA gene sequences with length longer than 200 bps in this study.

### Taxonomic classification and OTU assignment

The Ribosomal Database Project (RDP) classifier [29] was used to classify 16S rRNA pyrosequencing reads and to perform an operational taxonomic unit (OTU) assignment. After accomplishing the OTU assignment with a threshold of 80 % confidence, we carried out a further filtering step by removing operational taxonomic units (OTUs) that appeared in fewer than half of the total time points.

### Relative abundance analysis

To estimate the relative difference between individual OTUs at each time point, we calculated the relative abundance of individual OTUs. First, we defined the pyrosequencing read number of the *i*^*th*^ OTU at *j*^*th*^ time point as *Y*_*ij*_. Second, we defined the sum of the pyrosequencing read numbers at *j*^*th*^ time point as *Y*_*j*_. Finally, we divided *Y*_*ij*_ by *Y*_*j*_ to obtain the relative proportion *X*_*ij*_ of the *i*^*th*^ OTU at *j*^*th*^ time point_._ After all pyrosequencing read numbers were transformed into relative proportions, we removed the *l*^*th*^ OTU if both the maximum relative proportion of all *X*_*lj*_ was less than 0.1 and the coefficient of variation of all *X*_*lj*_ was larger than 3.4. In addition, we filtered out the data at the *k*^*th*^ time point if both the maximum of all *X*_*ik*_ was less than 0.1 and the total number of OTUs was less than 60 % at the *k*^*th*^ time point. Finally, the missing values were filled through the Bayesian principal component analysis (BPCA) with K = 5 [30].

### Data standardization

The relative proportion of each OTU at each time point was standardized in order to estimate the difference between OTUs at each time point. The relative proportion *X*_*ij*_ of the *i*^*th*^ OTU at the *j*^*th*^ time point was transformed such that it fell within the interval [0,1]. We defined *P*_*ij*_ as (*X*_*ij*_ − *X*_min_) /(*X*_max_ − *X*_min_), where *P*_*ij*_ is the standardized relative proportion (SRP) of the *i*^*th*^ OTU at the *j*^*th*^ time point, while *X*_min_ and *X*_max_ are the minimum and maximum relative proportions respectively.

### Network construction

**Fig. 1:**
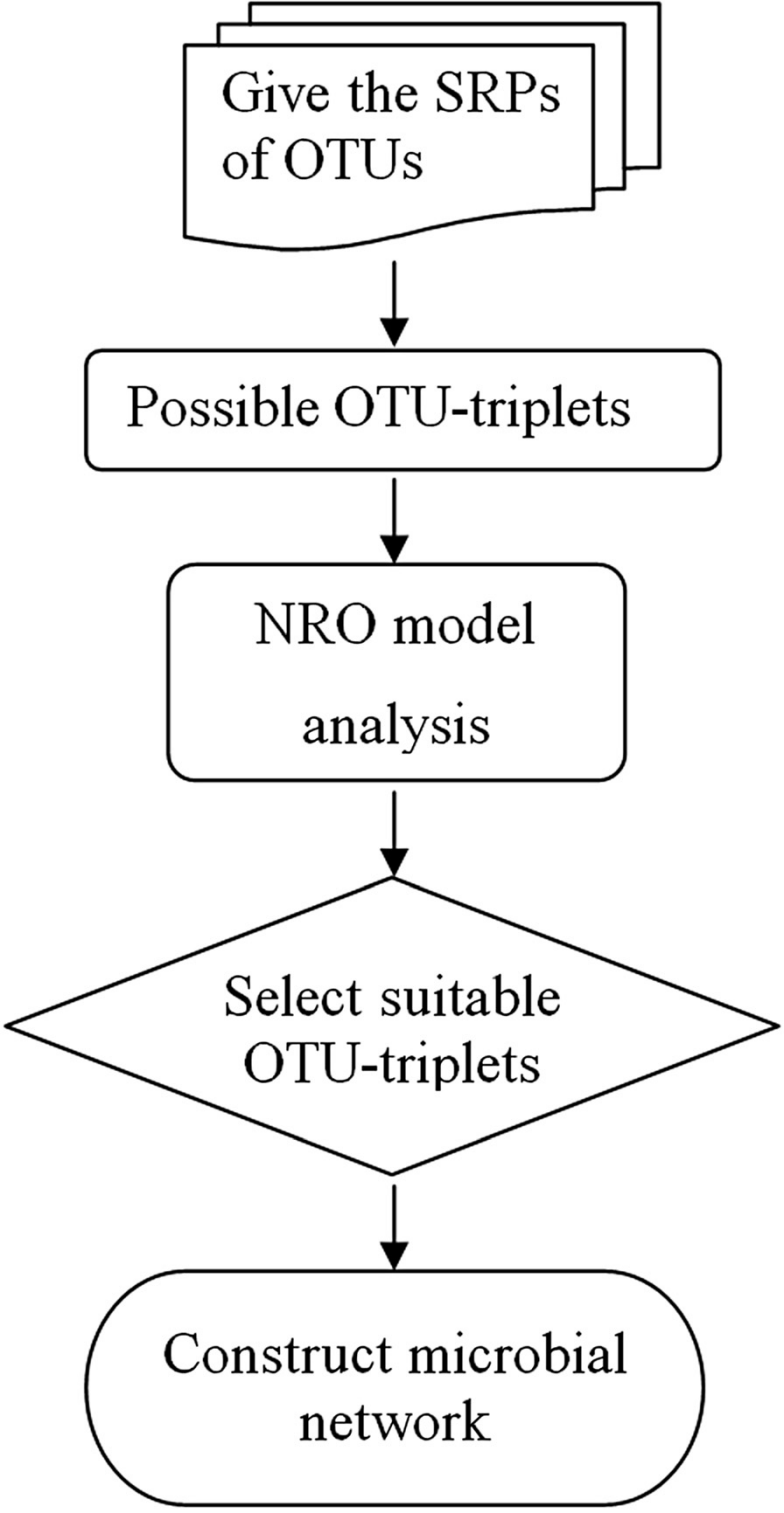
The flowchart of the RMN algorithm. First, the standardized relative proportions (SRPs) of OTUs were analyzed and found out possible OTU-triplets. Then, suitable OTU-triplets were selected by nonlinear regulatory OTU-triplet (NRO) model. Finally, the microbial network was reconstructed

**Fig. 2:**
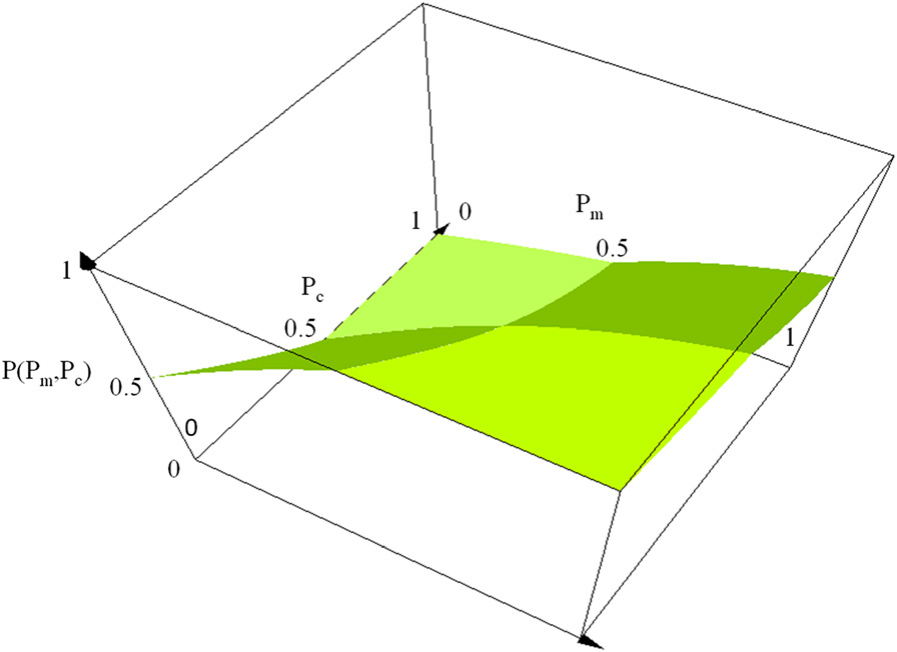
The 3D image of a three-dimensional model surface (3D-MS). *P*(*P*_*m,*_ *P*_*c*_) is the inferred the standardized relative proportion (SPR) of *O*_*t*_, where *P*_*m*_ and *P*_*c*_ are the SRPs of *O*_*m*_ and *O*_*c*_, respectively

We developed a rule-based microbial network (RMN) algorithm to construct microbial regulatory networks. The RMN algorithm is based on a nonlinear regulatory OTU-triplet model, named the NRO model for short. Figure 1 shows the flowchart of the RMN algorithm, and the details are as follows:

(i) To investigate how each OTU was involved in microbial regulatory network, we constructed a nonlinear regulatory OTU-triplet model by using triplets of OTUs across all time points (or samples). The nonlinear regulatory OTU-triplet model has three assumptions: First, a cooperation–competition system is assumed to exist in the microbial regulatory network. An OTU-triplet is defined as {*O*_*m*_, *O*_*c*_, *O*_*t*_}, where *O*_*m*_*, O*_*c*_, and *O*_*t*_ represent the *m*^*th*^ , *c*^*th*^ and *t*^*th*^ OTUs respectively. Here, *O*_*m*_ and *O*_*c*_ are assumed to be the cooperator and the competitor of *O*_*t*_ respectively. In other words, the relationship between the *m*^*th*^ and the *t*^*th*^ OTUs is assumed to be cooperative, while that between the *c*^*th*^ and the *t*^*th*^ OTUs is assumed to be competitive. Note that *O*_*m*_, *O*_*c*_ and *O*_*t*_ represent different microbes in genus level. Second, it is assumed that the standardized relative proportion (SRP) of *O*_*t*_ is high when the SRP of *O*_*m*_ is high and the SRP of *O*_*c*_ is low. Conversely, the SRP of *O*_*t*_ is assumed to be low when the SRP of the *O*_*m*_ is low and the SRP of the *O*_*c*_ is high. Third, the relationship between *O*_*m*_, *O*_*c*_ and *O*_*t*_ can be modeled by using a three-dimensional model surface (3D-MS) (Fig. 2) which is based on a piecewise nonlinear-quadratic polynomial:

1 $$ P\left({P}_m,{P}_c\right)=\left\{\begin{array}{l}2 \tanh \left(1.1{P}_m\right)\left(1- \tanh \left(1.1{P}_c\right)\right), \kern0.5em  if\ 0\le {P}_m<0.5\kern0.5em  and\kern0.5em 0.5<{P}_c\le 1;\\ {}1-2\left(1- \tanh \left(1.1{P}_m\right)\right) \tanh \left(1.1{P}_c\right), \kern0.5em  if\ 0.5<{P}_m\le 1\kern0.5em  and\kern0.5em 0\le {P}_c<0.5;\\ {} \tanh \left(1.1{P}_m\right)- \tanh \left(1.1{P}_c\right)+0.5, \kern0.5em  otherwise\end{array}\right. $$

In (1), *P*_*m*_ and *P*_*c*_ are the standardized relative proportions (SRPs) of *O*_*m*_ and *O*_*c*_ respectively while *P*(*P*_*m,*_*P*_*c*_) is the inferred SRP of *O*_*t*_.

(ii) To find suitable OTU-triplets close to the 3D-MS for the OTU-triplet regulatory model, we used a lack-of-fit function to analyze all possible OTU-triplets. The lack-of-fit function is defined as:

2 $$ L\left({O}_m,{O}_c,{O}_t\right)=\frac{{\displaystyle {\sum}_{n=1}^k\Big({P}_{tn}-}{P^{\hat{\mkern6mu}}}_{tn}\Big){}^2}{{\displaystyle {\sum}_{n=1}^k\Big({P}_{tn}-}{P^{-}}_{tn}\Big){}^2} $$

where *P*^*^*^_*tn*_ is the inferred *SRP* of *O*_*t*_ at the *n*^*th*^ time point (e.g. *P*(*P*_*m,*_*P*_*c*_) at the *n*^*th*^ time point ); *P*_*tn*_ is the observed SRP of *O*_*t*_ at the *n*^*th*^ time point; and *P*^−^_*tn*_ represents the average of *P*_*tn*_ across all *k* time points. A small *L*(*O*_*m*_, *O*_*c*_, *O*_*t*_) value indicates a good fit between an OTU-triplet {*O*_*m*_, *O*_*c*_, *O*_*t*_} and the 3D-MS. In other words, a smaller *L*(*O*_*m*_, *O*_*c*_, *O*_*t*_) value reveals a stronger relationship among *O*_*m*_, *O*_*c*_, and *O*_*t*_. Similar rationale has been successfully applied for constructing gene regulatory networks [24].

(iii) To check the reliability of OTU-triplets, we used an adjustment function to fix imperfect measurements at one or more time points (or samples). The adjustment function is defined as:

3 $$ D\left({O}_m,{O}_c,{O}_t\right)={\displaystyle {\sum}_{n=1}^k\left|\left(\frac{L_{(n)}\left({O}_m,{O}_c,{O}_t\right)}{L\left({O}_m,{O}_c,{O}_t\right)}\right){ \log}_{10}\left(\frac{L_{(n)}\left({O}_m,{O}_c,{O}_t\right)}{L\left({O}_m,{O}_c,{O}_t\right)}\right)\right|} $$

where *L*_*(n)*_(*O*_*m*_, *O*_*c*_, *O*_*t*_) is the value of lack-of-fit function when the information from the *n*^*th*^ time point is removed. A smaller *D*(*O*_*m*_, *O*_*c*_, *O*_*t*_) value indicates the information of the OTU-triplet relationship is reliable. Finally, an integrated function, *I*(*O*_*m*_, *O*_*c*_, *O*_*t*_) is defined as the value of *L*(*O*_*m*_, *O*_*c*_, *O*_*t*_) multiplied by the value of (1+ *D*(*O*_*m*_, *O*_*c*_, *O*_*t*_)). A smaller *I*(*O*_*m*_, *O*_*c*_, *O*_*t*_) value indicates a stronger OTU-triplet relationship among *O*_*m*_, *O*_*c*_, and *O*_*t.*_

(iv) Finally, we defined two criteria, *L*_*d*_(*O*_*m*_, *O*_*c*_, *O*_*t*_) = *L*(*O*_*m*_, *O*_*c*_, *O*_*t*_)/(1- *L*(*O*_*m*_, *O*_*c*_, *O*_*t*_)) and *D*(*O*_*m*_, *O*_*c*_, *O*_*t*_), to filter out unreliable OTU-triplets, when 0 ≦ *L*_*d*_(*O*_*m*_, *O*_*c*_, *O*_*t*_) ≦ *L* and *D*(*O*_*m*_, *O*_*c*_, *O*_*t*_) < *D*. Note the L and D are constants. We then ranked reliable OTU-triplets basing on the sorted *I*(*O*_*m*_, *O*_*c*_, *O*_*t*_) values in the ascending order, and selected OTU-triplets with lower *I*(*O*_*m*_, *O*_*c*_, *O*_*t*_) values as candidates for network construction.

### Performance analysis

To evaluate the performance of the RMN algorithm, we simulated a microbial regulatory network consisting of ten gut bacteria, three latent factors, and ten noise factors as follows:

4 $$ \begin{array}{l}{O}_1(s)=1.3 \tanh \left(-0.1{F}_2(s)+0.25\right)+{N}_1(s)\\ {}{O}_2(s)=1.1 \tanh \left(0.35{F}_2(s)+0.1\right)+{N}_2(s)\\ {}{O}_3(s)= \tanh \left(-0.56{O}_1(s)+0.4{F}_1(s)+0.5\right)+{N}_3(s)\\ {}{O}_4(s)= \tanh \left(0.6{O}_1(s)-0.15{O}_2(s)-0.1{O}_3(s)+0.1\right)+{N}_4(s)\\ {}{O}_5(s)= \tanh \left(0.24{O}_2(s)-0.75{O}_3(s)-0.05{F}_1(s)+0.3\right)+{N}_5(s)\\ {}{O}_6(s)=1.4 \tanh \left(0.2{O}_5(s)-0.2{O}_3(s)+0.15\right)+{N}_6(s)\\ {}{O}_7(s)=1.3 \tanh \left(-0.15{O}_5(s)+0.2\right)+{N}_7(s)\\ {}{O}_8(s)= \tanh \left(-0.15{F}_3(s)+0.2\right)+{N}_8(s)\\ {}{O}_9(s)= \tanh \left(-0.41{O}_6(s)+0.55{O}_7(s)+0.35{O}_8(s)+0.18\right)+{N}_9(s)\\ {}{O}_{10}(s)= \tanh \left(-0.45{O}_7(s)+0.7{O}_8(s)+0.1{F}_3(s)+0.25\right)+{N}_{10}(s)\end{array} $$

**Fig. 3:**
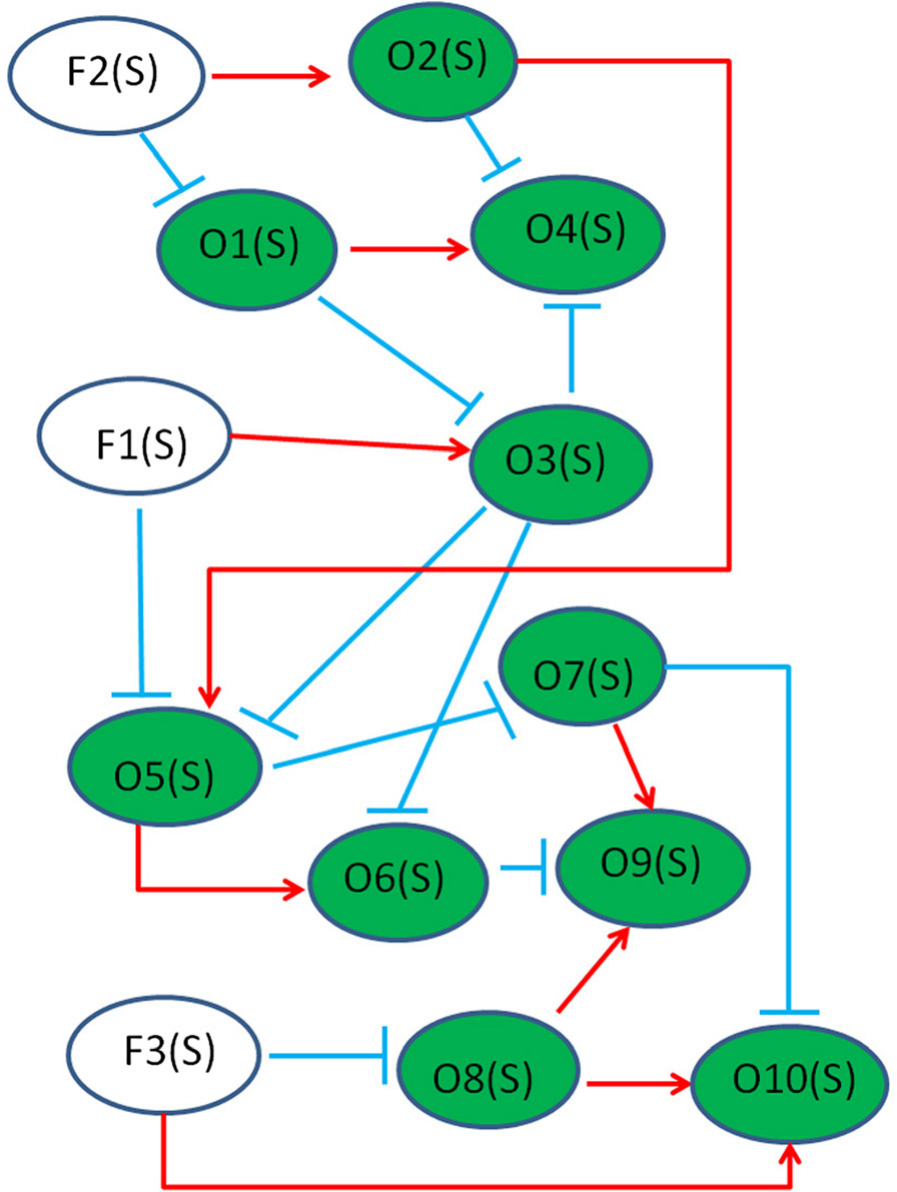
Simulation of microbial regulatory network. For example, *O*_*1*_(*s*) is defined to interact competitively with *O*_*3*_(*s*) but interact cooperatively with *O*_*4*_(*s*). Here, the competitive and cooperatively relationships are defined as ┤ and →, individually

We used Tanh functions to create simulated a microbial regulatory network because Tanh functions have been used to model regulatory responses of pathogens and microbes [25–27]. In addition, the similar idea has been used to create simulated gene networks for evaluating the performance of GASA method [31]. In detail, we defined this synthetic regulatory model in Fig. 3, where *O*_*i*_(*s*) is the abundance proportion of the *i*^*th*^ OTU with i =1,2,…,10; *F*_*j*_(*s*) is the abundance proportion of *j*^*th*^ latent factor with j = 1, 2, 3; and *N*_*k*_(*s*) is the abundance proportion of the *k*^*th*^ noise factor with k = 1,2,…,10. Note the value of *O*_*i*_(*s*) is between 0 and 1, while *F*_*j*_(*s*) is a random variable between 0 and 1. *N*_*k*_(*s*) is a random variable representing the noise level in the simulated data (when *N*_*k*_(*s*) = 0, no noise exists in the simulated data). Three noise levels are assumed in the simulated data: low noise if the value of *N*_*k*_(*s*) is equal to the variance of *O*_*i*_(*s*) divided by *R*_*k*_(*s*); medium noise if *N*_*k*_(*s*) is equal to five times the low noise level; and high noise if *N*_*k*_(*s*) is equal to ten times the low noise level. Here, *R*_*k*_(*s*) represents the signal-noise ratio and its range was between 4 and 12. Note that *R*_*k*_(*s*) is an integral and is generated by using time-series data of gut bacteria [19]. In detail, *R*_*k*_(*s*) is the average proportion of gut bacteria divided by its standard deviation.

We evaluated the performance of the RMN algorithm by calculating the following measures: true positive rate (TPR), true negative rate (TNR), F-measure, and Accuracy. TPR is defined as TP/(TP + FN), where TP and FN are the numbers of true positive interactions (links) and false negative interactions (links) respectively. TNR is defined as TN/(TN + FP), where TN and FP are the numbers of true negative interactions (links) and false positive interactions (links) respectively. We define F-measure as 2*TPR*TNR/ (TPR + TNR), and accuracy as (TP + TN)/(TP + FN + TN + FP).

## Results and discussion

### Performance of RMN algorithm using simulation data

**Fig. 4:**
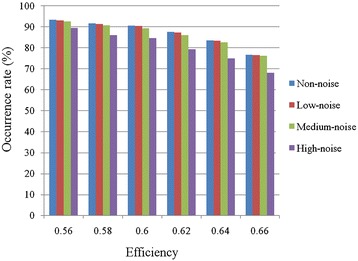
Efficiency analysis of RMN algorithm for simulated data under different level of noise interference. The threshold was set at 0≦*L*_*d*(_ *O*_*m*_, *O*_*c*_, *O*_*t*_) ≦0.8 and *D*(*O*_*m*_, *O*_*c*_, *O*_*t*_) < 0.8. Any three of four evaluation measures showed the efficiency are their ratio value superior or equal. The estimated data used 5000 simulated microbial regulatory networks. Each simulated data contains 10 OTUs, whose abundance values were simulated for 13 time points. Non-noise: simulated data without noise; Low-noise: simulated data with low noise; Medium-noise: simulated data with medium noise; High-noise: simulated data with high noise

**Fig. 5:**
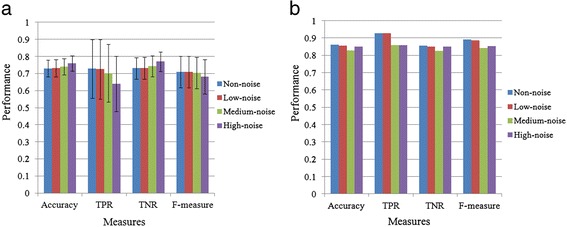
Evaluation measurements on efficiency analysis of RMN algorithm for simulated data under different level of noise interference. The threshold was set at 0≦*L*_*d*(_ *O*_*m*_, *O*_*c*_, *O*_*t*_) ≦0.8 and *D*(*O*_*m*_, *O*_*c*_, *O*_*t*_) < 0.8. **a: ** The average results of four evaluation measures for the efficiency of RMN algorithm. Error bars represent the standard deviation. **b:** The best result of four evaluation measures for the efficiency of RMN algorithm. The estimated data used 5000 simulated microbial regulatory networks. Each simulated data contains 10 OTUs, whose abundance values were simulated for 13 time points. Non-noise: simulated data without noise; Low-noise: simulated data with low noise; Medium-noise: simulated data with medium noise; High-noise: simulated data with high noise

We generated simulated data under different levels of noise and evaluated the performance of our RMN algorithm by using the above mentioned four evaluation measures. Then, we analyzed the occurrence rate (%) of 50000 simulated microbial regulatory networks when any three of four evaluation measures showed the efficiency superior or equal to the defined value. (see Fig. 4). All four evaluation measures suggest high performance from our RMN algorithm. Comparing different noise levels, RMN algorithm has shown the best efficiency when there is no noise in the simulated data. Even at medium noise level, over 82 % of the results show efficiency values higher than 0.64. In addition, the assessment from those four evaluation measures shows 0.64 efficiency value on more than 74 % simulated data. There are no significant differences between the performance results when the noise is varied from zero to medium level (see Fig. 5(a)). Although the performance of our RMN algorithm at the high noise level is worse than those from lower noise levels, its efficiency value is still over 0.68. More interestingly, according to TNR, there were fewer false positive interactions (links) when inferred from the high noise data than that from lower noise data (see Fig. 5(a)). In addition, best efficiency under different noise interferences exceeds 0.82 (see Fig. 5(b)). During developing RMN algorithm, we used two conditions, *L*_d_(*O*_m_, *O*_c_, *O*_t_) and *D*(*O*_m_, *O*_c_, *O*_t_), to filter out unreliable OTU-triplets, and we tried to select the suitable thresholds for *L*_d_(*O*_m_, *O*_c_, *O*_t_) and *D*(*O*_m_, *O*_c_, *O*_t_) by analyzing the simulation data (Table 1). The best efficiency for all evaluations reached over 0.82 when the threshold at *L*_*d*_(*O*_*m*_, *O*_*c*_, *O*_*t*_) and *D*(*O*_*m*_, *O*_*c*_, *O*_*t*_) were used (see Table 1). Moreover, the efficiency for all evaluations reached over 0.71 when the threshold at *L*_*d*_(*O*_*m*_, *O*_*c*_, *O*_*t*_) and *D*(*O*_*m*_, *O*_*c*_, *O*_*t*_). All in all, our efficiency evaluations demonstrate that the performance of our RMN algorithm is robust under three latent factors and different noise levels.

**Table 1:** The best performance result with different parameters and different level of noise interference. Here L0.8 represents for 0≦ *L*_*d*(_ *O*_*m*_, *O*_*c*_, *O*_*t*_) ≦0.8 and D0.8 represents for *D*(*O*_*m*_, *O*_*c*_, *O*_*t*_) < 0.8

Non-noise	Accuracy	TPR	TNR	F-measure
L0.8D0.8	0.861	0.929	0.855	0.890
L1.8D0.8	0.833	0.857	0.831	0.844
L2.8D0.8	0.756	0.857	0.747	0.798
L3.8D0.8	0.722	0.786	0.717	0.750
L0.8D1.8	0.822	0.857	0.819	0.838
L0.8D2.8	0.828	0.857	0.825	0.841
L0.8D3.8	0.828	0.857	0.825	0.841
Low-noise	Accuracy	TPR	TNR	F-measure
L0.8D0.8	0.856	0.929	0.849	0.887
L1.8D0.8	0.767	0.786	0.765	0.775
L2.8D0.8	0.756	0.786	0.753	0.769
L3.8D0.8	0.756	0.714	0.759	0.736
L0.8D1.8	0.828	0.857	0.825	0.841
L0.8D2.8	0.811	0.857	0.807	0.831
L0.8D3.8	0.822	0.857	0.819	0.838
Medium-noise	Accuracy	TPR	TNR	F-measure
L0.8D0.8	0.828	0.857	0.825	0.841
L1.8D0.8	0.811	0.786	0.813	0.799
L2.8D0.8	0.767	0.786	0.765	0.775
L3.8D0.8	0.761	0.786	0.759	0.772
L0.8D1.8	0.828	0.857	0.825	0.841
L0.8D2.8	0.828	0.857	0.825	0.841
L0.8D3.8	0.833	0.929	0.825	0.874
High-noise	Accuracy	TPR	TNR	F-measure
L0.8D0.8	0.850	0.857	0.849	0.853
L1.8D0.8	0.800	0.786	0.801	0.793
L2.8D0.8	0.756	0.786	0.753	0.769
L3.8D0.8	0.756	0.786	0.753	0.769
L0.8D1.8	0.833	0.857	0.831	0.844
L0.8D2.8	0.833	0.857	0.831	0.844
L0.8D3.8	0.844	0.857	0.843	0.850

### Inferring the regulatory relationship of microbial pairs with low correlation coefficient

Recent study shows that correlations between the abundances of microbes do not imply microbial interactions [18]. In order to investigate whether OTU-triplets obtained by RMN algorithm are significantly correlated based on their correlation coefficient and whether RMN algorithm can find the regulator relationship behind standard correlation-based approaches, we analyzed the optimal resulting triplets with true positive links (filtered by RMN algorithm). Here we used Spearman's rank correlation coefficient for the estimation of nonlinear correlation coefficient. The Pr value represents a probability of RMN algorithm prediction for OTU pairs whose nonlinear correlation coefficient is less than 0.5. In detail, Pr value (%) was defined as (*O*_*m*_*O*_*t*__count + *O*_*c*_*O*_*t*_ _count) *100 %/Total_count, where *O*_*m*_*O*_*t*__count is the number of resulting triplets with a true positive link between cooperator OTU and target OTU pair whose nonlinear correlation coefficient is less than 0.5; *O*_*c*_*O*_*t*_ _count is the number of resulting triplets with a true positive link between competitor OTU and target OTU pair whose nonlinear correlation coefficient is greater than −0.5; Total count is the number of resulting triplets with a true positive link. As Table 2 shown, under different levels of noise inference, Pr values for most analyzed results were more than 15 %. That may imply the resulting triplets contain OTU pairs with low nonlinear correlation coefficient. For example, more than 15 % OTU pairs with low nonlinear correlation coefficient (less than 0.5) could be found when the threshold was set at 0≦*L*_*d*_(*O*_*m*_, *O*_*c*_, *O*_*t*_) ≦0.8 and *D*(*O*_*m*_, *O*_*c*_, *O*_*t*_) < 0.8. Based on the threshold, the performances of RMN algorithm were more than 0.82 (see Table 1). In addition, more than 20 % OTU pairs with low nonlinear correlation coefficient (less than 0.5) could be found when the threshold was set at 0≦*L*_*d*_(*O*_*m*_, *O*_*c*_, *O*_*t*_) ≦3.8 and *D*(*O*_*m*_, *O*_*c*_, *O*_*t*_) < 0.8. Based on the threshold, the performances of RMN algorithm were more than 0.71 (see Table 1). In other words, the RMN algorithm may characterize the regulatory relationship composed of OTU pairs with low correlation coefficient, and has a choice to find the regulator relationship behind standard correlation-based approaches.

**Table 2:** The nonlinear correlation coefficient of OTU pairs, predicted by RMN algorithm as co-regulated triplets, under different level of noise interference. A triplet predicted by RMN algorithm is composed of cooperator, competitor and target. Here L0.8 represents for 0≦ *L*_*d*(_ *O*_*m*_, *O*_*c*_, *O*_*t*_) ≦0.8 and D0.8 represents for *D*(*O*_*m*_, *O*_*c*_, *O*_*t*_) < 0.8

Non-noise	*O*_m_ *O*_t__count	*O*_c_ *O*_t__count	Total_count	Pr value (%)
L0.8D0.8	3	3	26	23.1
L1.8D0.8	4	0	24	16.7
L2.8D0.8	7	5	24	50.0
L3.8D0.8	6	1	22	31.8
L0.8D1.8	2	2	24	16.7
L0.8D2.8	1	2	24	12.5
L0.8D3.8	1	1	24	8.3
Low-noise	*O*_m_ *O*_t__count	*O*_c_ *O*_t__count	Total_count	Pr value (%)
L0.8D0.8	3	1	26	15.4
L1.8D0.8	6	2	22	36.4
L2.8D0.8	5	0	22	22.7
L3.8D0.8	1	4	24	20.8
L0.8D1.8	4	2	24	25.0
L0.8D2.8	1	0	24	4.2
L0.8D3.8	3	1	24	16.7
Medium-noise	*O*_m_ *O*_t__count	*O*_c_ *O*_t__count	Total_count	Pr value (%)
L0.8D0.8	5	2	24	29.2
L1.8D0.8	5	3	22	36.4
L2.8D0.8	5	1	22	27.3
L3.8D0.8	7	1	22	36.4
L0.8D1.8	4	1	24	20.8
L0.8D2.8	2	1	24	12.5
L0.8D3.8	4	2	26	23.1
High-noise	*O*_m_ *O*_t__count	*O*_c_ *O*_t__count	Total_count	Pr value (%)
L0.8D0.8	3	3	24	25.0
L1.8D0.8	6	5	22	50.0
L2.8D0.8	7	4	22	50.0
L3.8D0.8	5	0	20	25.0
L0.8D1.8	3	2	24	20.8
L0.8D2.8	2	1	24	12.5
L0.8D3.8	2	2	24	16.7

### The construction of microbial regulatory network from infant gut data

To further demonstrate the capability of our RMN algorithm, we constructed microbial interaction networks from a set of real microbial community time series data. The data used consist of 61 infant gut samples treated with four diet states at four time periods [28].

**Fig. 6:**
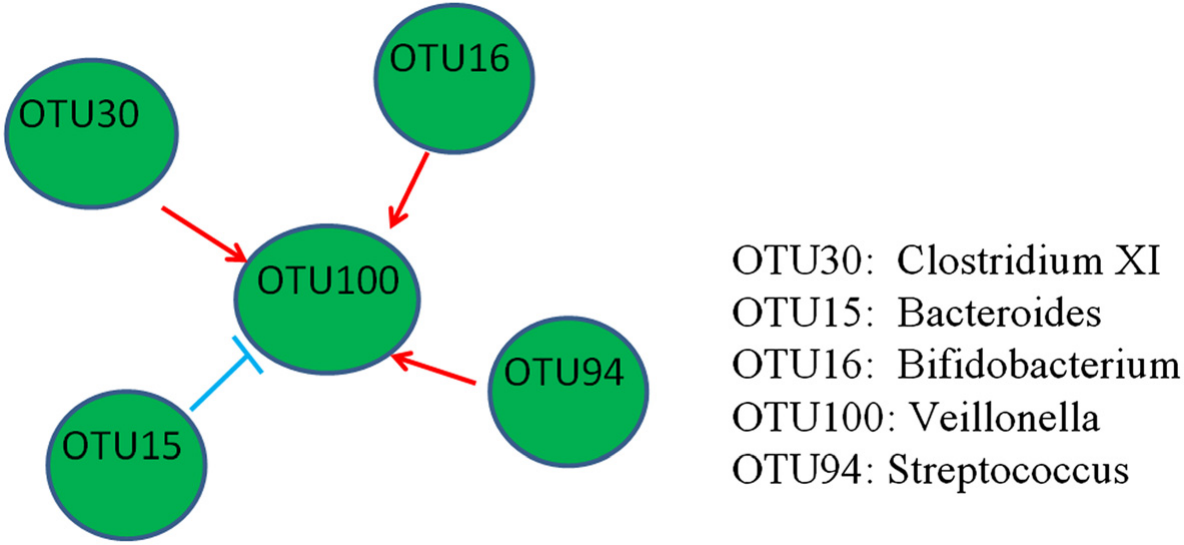
The reconstruction of microbial regulatory networks in infant intestinal tract. Here, the competitive and cooperatively relationships are defined as ┤and →, individually

After performing taxonomic classification and OTU assignment, 101 OTUs were obtained (Additional file 1). However, after the filtering process removing OTUs that appeared in less than half of the total time points, only 14 OTUs met the requirement (Additional file 2). In addition, after performing relative abundance analysis and filling missing values, 10 OTUs with 43 time points were obtained. Finally, according to Fig. 3c from Koenig, et al. [28], only 10 OTUs with 36 time points between four steps were selected for analyzing using our RMN algorithm (Additional file 3). Consistently, among 10 OTUs, 4 OTUs (OTU15, OTU16, OTU34, and OTU94) were found to be involved in competitive and cooperative interactions in Freilich’s study [32]. Since microbiome data are usually enormous and inherently noisy, the threshold was set at 0≦*L*_*d*(_*O*_*m*_, *O*_*c*_, *O*_*t*_) ≦3.8 and *D*(*O*_*m*_, *O*_*c*_, *O*_*t*_) < 0.8 for better prediction sensitivity (see Table 3). Before filtering by this threshold, top 100 possible OTU-triplets from 10 OTUs analysis were listed in Additional file 4. After filtering by this threshold, only 3 reliable OTU-triplets from 5 OTUs (Additional file 5) were used to construct microbial regulatory network. The resulting microbial interaction network among five OTUs is displayed in Fig. 6. Gram-negative anaerobic cocci, *Veillonella* (OTU100), is found to interact cooperatively with three OTUs, namely *Clostridium XI* (OTU30), *Bifidobacterium* (OTU16) and *Streptococcus* (OTU94), but competitively with *Bacteroides* (OTU15). Among them, *Bifidobacterium*, *Streptococcus*, and *Bacteroides* have been identified in competitive and cooperative metabolic interactions [32]. In particular, we observed a positive correlation between *Veillonella* and *Bifidobacterium* , both residing in human intestines and oral mucosa as anaerobic commensal organisms [33]. This directional interaction also suggests the commensal relationship between *Veillonella* and *Bifidobacterium*. In addition, *Veillonella* was also observed to have a positive correlation with *Streptococcus*. They have been found to interact cooperatively during the production and degradation of Lactic acid [34–40]. Recently, positive correlations were also found between the abundances of *Veillonella* and *Streptococcus* [41]. In addition, the constructed network also suggests that competitive relationship may exist between *Veillonella* and *Bacteroides. Veillonella* and *Bacteroides* are gut-associated obligate anaerobic genera found in maternal faeces, breast milk and neonatal faeces [42]; and the difference in their efficiency on oligosaccharide consumptions may suggest they occupy different metabolic niches [43]. In addition, our network suggests there might be a cooperative relationship between *Veillonella* and *Clostridium XI Clostridium glycolicum*; and this cooperative relationship might suggest their simultaneous involvement in the ear infections in infants. In fact, those two gut bacteria have been found to be associated with bacterial infections, such as bacteremia [44, 45]. Finally, microbial interaction network from Fig. 6 were analyzed to check if the RMN algorithm can predict microbial interactions from low correlation data. As shown, it confirmed that OTU pairs with low correlations can be predicted by RMN algorithm (Additional file 6). In general, our constructed network can extrapolate cooperative and competitive relationships between microbes in infant guts.

**Table 3:** The efficiency analysis under different level of noise interference by using the simulated data who’s best performance showed at a threshold on 0≦ *L*_*d*(_ *O*_*m*_, *O*_*c*_, *O*_*t*_) ≦0.8 and *D*(*O*_*m*_, *O*_*c*_, *O*_*t*_) < 0.8

Non-noise	Accuracy	TPR	TNR	F-measure
L0.8D0.8	0.861	0.929	0.855	0.890
L1.8D0.8	0.706	1.000	0.681	0.810
L2.8D0.8	0.617	1.000	0.584	0.738
L3.8D0.8	0.556	1.000	0.518	0.683
L0.8D1.8	0.828	0.929	0.819	0.871
L0.8D2.8	0.828	0.929	0.819	0.871
L0.8D3.8	0.828	0.929	0.819	0.871
Low-noise	Accuracy	TPR	TNR	F-measure
L0.8D0.8	0.856	0.929	0.849	0.887
L1.8D0.8	0.678	0.929	0.657	0.769
L2.8D0.8	0.611	0.929	0.584	0.717
L3.8D0.8	0.550	0.929	0.518	0.665
L0.8D1.8	0.761	0.929	0.747	0.828
L0.8D2.8	0.761	0.929	0.747	0.828
L0.8D3.8	0.761	0.929	0.747	0.828
Medium-noise	Accuracy	TPR	TNR	F-measure
L0.8D0.8	0.828	0.857	0.825	0.841
L1.8D0.8	0.706	0.929	0.687	0.790
L2.8D0.8	0.622	0.929	0.596	0.726
L3.8D0.8	0.533	0.929	0.500	0.650
L0.8D1.8	0.744	0.857	0.735	0.791
L0.8D2.8	0.744	0.857	0.735	0.791
L0.8D3.8	0.744	0.857	0.735	0.791
High-noise	Accuracy	TPR	TNR	F-measure
L0.8D0.8	0.850	0.857	0.849	0.853
L1.8D0.8	0.733	0.929	0.717	0.809
L2.8D0.8	0.633	0.929	0.608	0.735
L3.8D0.8	0.572	0.929	0.542	0.685
L0.8D1.8	0.778	0.857	0.771	0.812
L0.8D2.8	0.778	0.857	0.771	0.812
L0.8D3.8	0.778	0.857	0.771	0.812

## Conclusions

In this paper we presented RMN algorithm, a rule-based algorithm using the OTU-triplet model with parametric weighting function, to construct microbial regulatory networks. RMN algorithm can theoretically extract directional interactions to delineate the cooperative and competitive relationships between microbes from high-throughput sequencing time series data. The efficiency estimation shows the high performance of RMN algorithm on simulated data with three latent factors and different noise levels. We also applied our framework to identify new relationships in infant gut samples. RMN algorithm is both computationally feasible and capable of detecting biologically significant progresses embedded in a microbial community.

Although simple pairwise relationship has been used to describe complex forms of microbial interactions, advanced techniques are still required to infer complex microbial networks. Recent studies show that the cooperative and competitive relationships between microbes coexist in complicated ecological communities [8, 22, 23]. In addition, triplet relation, like activator-repressor-target pattern, has been adopted to construct gene regulatory network form time course gene expression data [24]. Basing on such a rationale, we developed RMN algorithm to describe the relationship among every triplet of microbe as the cooperator-competitor-target. RMN algorithm is used to search for triplet relations of OTUs where the abundances of the target should be high while that for the cooperator is high and that for the competitor is low (conversely, the abundances of the target should be low while that for the cooperator is low and that for the competitor is high). Our evaluation results suggest RMN algorithm has high performance when subjected to simulated data with different levels of noise (see Figs. 4 and 5). As presented in the results, our algorithm has characterized well-known and possible microbial interactions from time series microbiome data of infant gut samples (see Fig. 6).

In summary, networks reconstructed by using simple similarity-based method often fail to capture the complex topology of a real system, and such networks may be full of false positive links. Furthermore, biologically significant relationships are often difficult to be identified from such networks. Our RMN algorithm can produce low false positive links even for highly noisy data (see Fig. 5(a)). Moreover, RMN algorithm is designed to explore complex networks consisted of non-linear, decentralized and directed interactions among microbes. However, having said all these, the drawback of RMN algorithm is that it may not be able to detect simple linear correlation between two OTUs. Another potential concern is that we tested our method using a simulated data with the Tanh functions, the circularity in the model construction and validation can cause bias on our results. Overall, RMN algorithm can theoretically predict microbial interactions from low correlation data (see Tables 1 and 2). Our method should be a promising starting step to identify novel microbial interactions that normally cannot be found by using similarity-based methods. Therefore, by integrating similarity-based methods and our RMN algorithm, one can potentially gain a more accurate picture of microbial interactions towards a better understanding of microbial dynamics.

## Additional files

Additional file 1:
**The table of OTUs for all the samples at the genus level with OTU assignment before filtering process.** (XLS 81 kb) Additional file 2:
**The table of OTUs for all the samples at the genus level with OTU assignment after filtering process.** (XLS 34 kb) Additional file 3:
**The table of OTUs for all the samples at the genus level with data standardization.** (XLS 26 kb) Additional file 4:
**List of top 100 possible OTU-triplets ranking by** ***I*** **(** ***O***_**m**_ **,** ***O***_**c**_ **,** ***O***_**t**_ **) values in the ascending order.** These OTU-triplets were selected without using the threshold set at 0≦*L*_d_(*O*_m_, *O*_c_, *O*_t_) ≦3.8 and *D*(*O*_m_, *O*_c_, *O*_t_) < 0.8. (XLS 28 kb) Additional file 5:
**List of the reliable OTU-triplets ranking by** ***I*** **(** ***O***_**m**_ **,** ***O***_**c**_ **,** ***O***_**t**_ **) values in the ascending order.** These OTU-triplets were selected when the threshold was set at 0≦*L*_d_(*O*_m_, *O*_c_,* O*_t_) ≦3.8 and *D*(*O*_m_, *O*_c_, *O*_t_) < 0.8. (XLS 19 kb) Additional file 6:
**The nonlinear correlation coefficient of OTU pairs predicted by RMN algorithm.** (XLS 20 kb)

## Abbreviations

RMN: Rule-based microbial network
SRS: Smooth response surface
3D-MS: Three-dimensional model surface
NRO: Nonlinear regulatory OTU-triplet
RDP: Ribosomal Database Project
OTU: Operational taxonomic unit
OTUs: Operational taxonomic units
BPCA: Bayesian principal component analysis
SRP: Standardized relative proportion
SRPs: Standardized relative proportions
TPR: True positive rate
TNR: True negative rate
